# 1-(4-*tert*-Butyl­benz­yl)-3-phenyl-1*H*-pyrazole-5-carboxylic acid

**DOI:** 10.1107/S1600536809017000

**Published:** 2009-05-14

**Authors:** Zheng Tang, Xiao-Ling Ding, Yong-Sheng Xie, Bao-Xiang Zhao

**Affiliations:** a Submarine College of Navy, Qingdao 266071, People’s Republic of China; b College of Advanced Professional Technology, Qingdao University, Qingdao 266061, People’s Republic of China; c School of Chemistry and Chemical Engineering, Shandong University, Jinan 250100, People’s Republic of China

## Abstract

In the title compound, C_21_H_22_N_2_O_2_, the mean plane of the pyrazole ring makes dihedral angles of 18.80 (12) and 77.13 (5)°, respectively, with the mean planes of the phenyl and *tert*-butyl­benzyl rings. The carboxylate group is inclined at 8.51 (14)° with respect to the pyrazole ring. The crystal structure displays inter­molecular O—H⋯O hydrogen bonding, generating centrosymmetric dimers.

## Related literature

For the synthesis and biological activity of related compounds, see: Wei *et al.* (2006 ▶); Xia *et al.* (2007*b* ▶); Zhang *et al.* (2008 ▶); Zhao *et al.* (2008 ▶). For related structures, see: Ding *et al.* (2007 ▶); Tang *et al.* (2007 ▶); Xia *et al.* (2007*a* ▶).
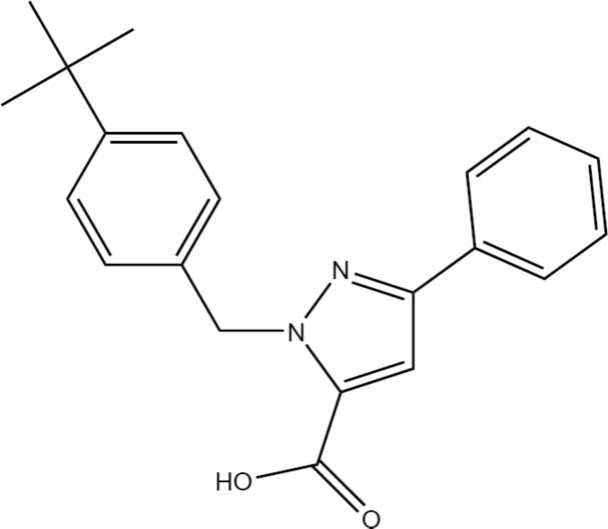


## Experimental

### 

#### Crystal data

C_21_H_22_N_2_O_2_
*M* *_r_* = 334.41
Monoclinic, 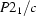

*a* = 12.336 (2) Å
*b* = 17.632 (3) Å
*c* = 8.7876 (17) Å
β = 97.910 (3)°
*V* = 1893.2 (6) Å^3^
*Z* = 4
Mo *K*α radiation
μ = 0.08 mm^−1^
*T* = 298 K
0.16 × 0.13 × 0.10 mm

#### Data collection

Bruker SMART CCD area-detector diffractometer
Absorption correction: multi-scan (*SADABS*; Bruker, 2005 ▶) *T*_min_ = 0.988, *T*_max_ = 0.992
10007 measured reflections
3552 independent reflections
2644 reflections with *I* > 2σ(*I*)
*R*_int_ = 0.021

#### Refinement

*R*[*F* ^2^ > 2σ(*F* ^2^)] = 0.052
*wR*(*F* ^2^) = 0.166
*S* = 1.02
3552 reflections
231 parameters
H-atom parameters constrained
Δρ_max_ = 0.56 e Å^−3^
Δρ_min_ = −0.27 e Å^−3^

### 

Data collection: *SMART* (Bruker, 2005 ▶); cell refinement: *SAINT* (Bruker, 2005 ▶); data reduction: *SAINT*; program(s) used to solve structure: *SHELXS97* (Sheldrick, 2008 ▶); program(s) used to refine structure: *SHELXL97* (Sheldrick, 2008 ▶); molecular graphics: *XP* in *SHELXTL* (Sheldrick, 2008 ▶); software used to prepare material for publication: *SHELXL97*.

## Supplementary Material

Crystal structure: contains datablocks I, global. DOI: 10.1107/S1600536809017000/pv2149sup1.cif

Structure factors: contains datablocks I. DOI: 10.1107/S1600536809017000/pv2149Isup2.hkl

Additional supplementary materials:  crystallographic information; 3D view; checkCIF report

## Figures and Tables

**Table 1:** Hydrogen-bond geometry (Å, °)

*D*—H⋯*A*	*D*—H	H⋯*A*	*D*⋯*A*	*D*—H⋯*A*
O1—H1⋯O2^i^	0.82	1.82	2.641 (2)	178

Symmetry code: (i) 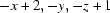.

**Fig. 1.**
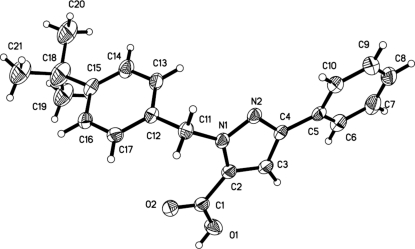
The molecular structure of (I), with displacement ellipsoids drawn at the 30% probability level.

## supplementary crystallographic information

### Comment

The pyrazole unit is one of the core structures in a number of natural products. Many pyrazole derivatives are known to exhibit a wide range of biological properties such as antitumor (Wei *et al.*, 2006). As a part of our continuing project on the study of synthesis and bioactivity evaluation of pyrazole derivatives (Xia *et al.*, 2007*b*; Zhao *et al.*, 2008; Zhang *et al.*, 2008), we report here the synthesis and crystal structure of the title compound.

In the title compound (Fig. 1), the pyrazole ring makes dihedral angles of 18.80 (12) and 77.13 (5)° with the phenyl and *tert*-butylbenzyl rings, respectively. The oxalate group is inclined at 8.51 (14)° with respect to the pyrazole ring. The crystal structure displays a strong intermolecular interaction which leads to the formation of hydrogen bonded dimeric units (Table 1) about inversion centers which is typical of organic carboxylic acids (Ding *et al.*, 2007). The crystal structures of a few related compounds have been reported from our laboratory, e.g. (Ding *et al.*, 2007; Xia *et al.*, 2007*a*; Tang *et al.*, 2007)

### Experimental

A mixture of ethyl 1-(4-*tert*-Butylbenzyl)-3-phenyl-1*H*-pyrazole-5-carboxylate (0.01 mol) and potassium hydroxide (0.02 mol) in ethanol (40 ml) was heated to reflux for 3 h. The solvent was removed under reduced pressure and the residue was dissolved in water and acidified with hydrochloric acid (10%). The precipitate was filtered and dried to give a white solid (yield 92%). Crystals of (I) suitable for X-ray diffraction were obtained by slow evaporation of a solution of the solid in acetone at room temperature for 3 d.

### Refinement

All H atoms were placed in calculated positions,with O—H = 0.82 Å and C—H = 0.93–0.97 Å, and included in the final cycles of refinement using a riding model, with *U*_iso_(H) = 1.2U_eq_(C) for aryl and methylene H atoms or 1.5U_eq_(C/O) for methyl and hydroxyl H atoms.

### Crystal data

**Table:**

C_21_H_22_N_2_O_2_	*F*(000) = 712
*M**_r_* = 334.41	*D*_x_ = 1.173 Mg m^−^^3^
Monoclinic, *P*2_1_/*c*	Mo *K*α radiation, λ = 0.71073 Å
Hall symbol: -P 2ybc	Cell parameters from 3769 reflections
*a* = 12.336 (2) Å	θ = 2.3–27.1°
*b* = 17.632 (3) Å	µ = 0.08 mm^−^^1^
*c* = 8.7876 (17) Å	*T* = 298 K
β = 97.910 (3)°	Block, colourless
*V* = 1893.2 (6) Å^3^	0.16 × 0.13 × 0.10 mm
*Z* = 4	

### Data collection

**Table:**

Bruker SMART CCD area-detector diffractometer	3552 independent reflections
Radiation source: fine-focus sealed tube	2644 reflections with *I* > 2σ(*I*)
graphite	*R*_int_ = 0.021
φ and ω scans	θ_max_ = 26.1°, θ_min_ = 2.0°
Absorption correction: multi-scan (*SADABS*; Bruker, 2005)	*h* = −12→15
*T*_min_ = 0.988, *T*_max_ = 0.992	*k* = −21→21
10007 measured reflections	*l* = −10→7

### Refinement

**Table:**

Refinement on *F*^2^	Secondary atom site location: difference Fourier map
Least-squares matrix: full	Hydrogen site location: inferred from neighbouring sites
*R*[*F*^2^ > 2σ(*F*^2^)] = 0.052	H-atom parameters constrained
*wR*(*F*^2^) = 0.166	*w* = 1/[σ^2^(*F*_o_^2^) + (0.0935*P*)^2^ + 0.4012*P*] where *P* = (*F*_o_^2^ + 2*F*_c_^2^)/3
*S* = 1.02	(Δ/σ)_max_ = 0.001
3552 reflections	Δρ_max_ = 0.56 e Å^−^^3^
231 parameters	Δρ_min_ = −0.27 e Å^−^^3^
0 restraints	Extinction correction: *SHELXL97* (Sheldrick, 2008), Fc^*^=kFc[1+0.001xFc^2^λ^3^/sin(2θ)]^-1/4^
Primary atom site location: structure-invariant direct methods	Extinction coefficient: 0.0065 (19)

### Special details

**Table:**

Geometry. All esds (except the esd in the dihedral angle between two l.s. planes) are estimated using the full covariance matrix. The cell esds are taken into account individually in the estimation of esds in distances, angles and torsion angles; correlations between esds in cell parameters are only used when they are defined by crystal symmetry. An approximate (isotropic) treatment of cell esds is used for estimating esds involving l.s. planes.
Refinement. Refinement of *F*^2^ against ALL reflections. The weighted *R*-factor *wR* and goodness of fit *S* are based on *F*^2^, conventional *R*-factors *R* are based on *F*, with *F* set to zero for negative *F*^2^. The threshold expression of *F*^2^ > σ(*F*^2^) is used only for calculating *R*-factors(gt) *etc*. and is not relevant to the choice of reflections for refinement. *R*-factors based on *F*^2^ are statistically about twice as large as those based on *F*, and *R*- factors based on ALL data will be even larger.

### Fractional atomic coordinates and isotropic or equivalent isotropic displacement parameters (Å^2^)

**Table:**

	*x*	*y*	*z*	*U*_iso_*/*U*_eq_	
O1	1.06981 (13)	0.07159 (8)	0.39610 (19)	0.0671 (4)	
H1	1.0670	0.0259	0.4125	0.101*	
O2	0.94281 (12)	0.07589 (8)	0.5566 (2)	0.0633 (4)	
N1	0.95215 (12)	0.23985 (8)	0.53903 (17)	0.0443 (4)	
N2	0.98423 (12)	0.31199 (8)	0.52234 (17)	0.0467 (4)	
C1	1.00463 (15)	0.10731 (11)	0.4774 (2)	0.0512 (5)	
C2	1.01498 (14)	0.19024 (10)	0.4691 (2)	0.0469 (4)	
C3	1.09088 (14)	0.23321 (11)	0.4044 (2)	0.0489 (4)	
H3	1.1445	0.2157	0.3484	0.059*	
C4	1.06969 (14)	0.30870 (10)	0.4412 (2)	0.0451 (4)	
C5	1.12601 (14)	0.37881 (11)	0.4041 (2)	0.0491 (5)	
C6	1.19031 (17)	0.38040 (14)	0.2862 (3)	0.0629 (5)	
H6	1.2004	0.3363	0.2318	0.075*	
C7	1.2401 (2)	0.44801 (18)	0.2487 (3)	0.0776 (7)	
H7	1.2830	0.4484	0.1695	0.093*	
C8	1.2262 (2)	0.51358 (16)	0.3277 (4)	0.0820 (8)	
H8	1.2583	0.5585	0.3012	0.098*	
C9	1.1646 (2)	0.51239 (14)	0.4457 (4)	0.0803 (8)	
H9	1.1556	0.5567	0.4999	0.096*	
C10	1.11504 (17)	0.44554 (12)	0.4858 (3)	0.0647 (6)	
H10	1.0745	0.4455	0.5675	0.078*	
C11	0.86035 (15)	0.22509 (11)	0.6259 (2)	0.0488 (4)	
H11A	0.8471	0.2703	0.6836	0.059*	
H11B	0.8810	0.1847	0.6991	0.059*	
C12	0.75498 (14)	0.20293 (10)	0.5253 (2)	0.0439 (4)	
C13	0.70381 (16)	0.25233 (12)	0.4155 (2)	0.0563 (5)	
H13	0.7353	0.2993	0.4014	0.068*	
C14	0.60599 (16)	0.23267 (14)	0.3261 (3)	0.0630 (6)	
H14	0.5739	0.2667	0.2525	0.076*	
C15	0.55480 (15)	0.16391 (13)	0.3434 (2)	0.0550 (5)	
C16	0.60596 (16)	0.11511 (12)	0.4557 (3)	0.0581 (5)	
H16	0.5734	0.0688	0.4718	0.070*	
C17	0.70455 (15)	0.13405 (11)	0.5443 (2)	0.0525 (5)	
H17	0.7371	0.0999	0.6174	0.063*	
C18	0.44732 (18)	0.14091 (16)	0.2427 (3)	0.0751 (7)	
C19	0.4710 (3)	0.0946 (3)	0.1148 (6)	0.185 (3)	
H19A	0.4903	0.0443	0.1506	0.277*	
H19B	0.5309	0.1165	0.0709	0.277*	
H19C	0.4076	0.0924	0.0383	0.277*	
C20	0.3864 (3)	0.2129 (3)	0.1715 (6)	0.151 (2)	
H20A	0.4274	0.2350	0.0975	0.227*	
H20B	0.3794	0.2490	0.2513	0.227*	
H20C	0.3150	0.1988	0.1219	0.227*	
C21	0.3666 (3)	0.1096 (3)	0.3438 (5)	0.148 (2)	
H21A	0.2932	0.1199	0.2967	0.222*	
H21B	0.3792	0.1334	0.4429	0.222*	
H21C	0.3767	0.0558	0.3553	0.222*	

### Atomic displacement parameters (Å^2^)

**Table:**

	*U*^11^	*U*^22^	*U*^33^	*U*^12^	*U*^13^	*U*^23^
O1	0.0727 (10)	0.0507 (8)	0.0822 (11)	0.0079 (7)	0.0261 (8)	0.0001 (7)
O2	0.0514 (8)	0.0513 (8)	0.0893 (11)	0.0027 (6)	0.0172 (8)	0.0011 (7)
N1	0.0354 (7)	0.0495 (8)	0.0459 (8)	−0.0024 (6)	−0.0012 (6)	−0.0039 (6)
N2	0.0380 (8)	0.0489 (8)	0.0510 (9)	−0.0027 (6)	−0.0009 (6)	−0.0040 (6)
C1	0.0404 (10)	0.0509 (10)	0.0596 (11)	0.0039 (8)	−0.0021 (8)	−0.0019 (8)
C2	0.0369 (9)	0.0495 (10)	0.0517 (10)	0.0034 (7)	−0.0025 (8)	−0.0008 (8)
C3	0.0362 (9)	0.0543 (11)	0.0551 (11)	0.0057 (7)	0.0024 (8)	0.0004 (8)
C4	0.0322 (8)	0.0537 (10)	0.0466 (9)	0.0018 (7)	−0.0045 (7)	0.0006 (7)
C5	0.0321 (9)	0.0558 (10)	0.0563 (11)	0.0025 (7)	−0.0055 (8)	0.0072 (8)
C6	0.0485 (11)	0.0788 (14)	0.0604 (12)	−0.0038 (10)	0.0045 (9)	0.0025 (10)
C7	0.0564 (13)	0.102 (2)	0.0754 (16)	−0.0117 (12)	0.0110 (11)	0.0205 (14)
C8	0.0559 (14)	0.0742 (17)	0.114 (2)	−0.0116 (11)	0.0055 (14)	0.0295 (15)
C9	0.0598 (14)	0.0591 (13)	0.123 (2)	−0.0048 (10)	0.0163 (14)	0.0034 (13)
C10	0.0493 (11)	0.0559 (12)	0.0906 (16)	−0.0013 (9)	0.0159 (11)	−0.0021 (11)
C11	0.0436 (10)	0.0590 (11)	0.0434 (9)	−0.0048 (8)	0.0043 (8)	−0.0049 (8)
C12	0.0363 (9)	0.0530 (10)	0.0430 (9)	−0.0008 (7)	0.0076 (7)	−0.0061 (7)
C13	0.0421 (10)	0.0633 (12)	0.0634 (12)	−0.0089 (8)	0.0069 (9)	0.0134 (9)
C14	0.0412 (10)	0.0838 (15)	0.0624 (13)	−0.0037 (9)	0.0016 (9)	0.0231 (11)
C15	0.0344 (9)	0.0760 (13)	0.0546 (11)	−0.0022 (8)	0.0062 (8)	−0.0029 (9)
C16	0.0435 (10)	0.0521 (11)	0.0772 (14)	−0.0076 (8)	0.0029 (9)	−0.0051 (9)
C17	0.0455 (10)	0.0488 (10)	0.0611 (12)	0.0011 (8)	−0.0004 (9)	0.0014 (8)
C18	0.0397 (11)	0.1038 (19)	0.0782 (16)	−0.0116 (11)	−0.0042 (10)	−0.0058 (13)
C19	0.072 (2)	0.270 (7)	0.196 (5)	0.018 (3)	−0.036 (3)	−0.159 (5)
C20	0.077 (2)	0.157 (4)	0.195 (5)	0.009 (2)	−0.067 (3)	−0.013 (3)
C21	0.0644 (19)	0.220 (5)	0.151 (4)	−0.052 (3)	−0.013 (2)	0.029 (3)

### Geometric parameters (Å, °)

**Table:**

O1—C1	1.308 (2)	C11—H11B	0.9700
O1—H1	0.8200	C12—C17	1.385 (3)
O2—C1	1.233 (3)	C12—C13	1.385 (3)
N1—N2	1.346 (2)	C13—C14	1.390 (3)
N1—C2	1.369 (2)	C13—H13	0.9300
N1—C11	1.473 (2)	C14—C15	1.385 (3)
N2—C4	1.352 (2)	C14—H14	0.9300
C1—C2	1.470 (3)	C15—C16	1.393 (3)
C2—C3	1.386 (3)	C15—C18	1.544 (3)
C3—C4	1.403 (3)	C16—C17	1.391 (3)
C3—H3	0.9300	C16—H16	0.9300
C4—C5	1.477 (3)	C17—H17	0.9300
C5—C6	1.389 (3)	C18—C19	1.451 (5)
C5—C10	1.395 (3)	C18—C21	1.525 (5)
C6—C7	1.401 (4)	C18—C20	1.561 (5)
C6—H6	0.9300	C19—H19A	0.9600
C7—C8	1.372 (4)	C19—H19B	0.9600
C7—H7	0.9300	C19—H19C	0.9600
C8—C9	1.367 (4)	C20—H20A	0.9600
C8—H8	0.9300	C20—H20B	0.9600
C9—C10	1.395 (3)	C20—H20C	0.9600
C9—H9	0.9300	C21—H21A	0.9600
C10—H10	0.9300	C21—H21B	0.9600
C11—C12	1.519 (2)	C21—H21C	0.9600
C11—H11A	0.9700		
			
C1—O1—H1	109.5	C17—C12—C11	121.19 (16)
N2—N1—C2	111.20 (15)	C13—C12—C11	120.99 (17)
N2—N1—C11	118.80 (15)	C12—C13—C14	120.89 (18)
C2—N1—C11	129.99 (16)	C12—C13—H13	119.6
N1—N2—C4	106.21 (14)	C14—C13—H13	119.6
O2—C1—O1	124.52 (19)	C15—C14—C13	122.00 (19)
O2—C1—C2	122.64 (18)	C15—C14—H14	119.0
O1—C1—C2	112.81 (18)	C13—C14—H14	119.0
N1—C2—C3	106.92 (16)	C14—C15—C16	116.70 (17)
N1—C2—C1	123.70 (17)	C14—C15—C18	122.4 (2)
C3—C2—C1	129.20 (17)	C16—C15—C18	120.9 (2)
C2—C3—C4	105.44 (17)	C17—C16—C15	121.59 (19)
C2—C3—H3	127.3	C17—C16—H16	119.2
C4—C3—H3	127.3	C15—C16—H16	119.2
N2—C4—C3	110.23 (16)	C12—C17—C16	121.04 (18)
N2—C4—C5	120.35 (16)	C12—C17—H17	119.5
C3—C4—C5	129.42 (18)	C16—C17—H17	119.5
C6—C5—C10	118.23 (19)	C19—C18—C21	117.7 (4)
C6—C5—C4	121.08 (19)	C19—C18—C15	110.1 (2)
C10—C5—C4	120.68 (18)	C21—C18—C15	109.8 (2)
C5—C6—C7	120.4 (2)	C19—C18—C20	106.5 (4)
C5—C6—H6	119.8	C21—C18—C20	102.2 (3)
C7—C6—H6	119.8	C15—C18—C20	110.2 (2)
C8—C7—C6	120.6 (2)	C18—C19—H19A	109.5
C8—C7—H7	119.7	C18—C19—H19B	109.5
C6—C7—H7	119.7	H19A—C19—H19B	109.5
C9—C8—C7	119.5 (2)	C18—C19—H19C	109.5
C9—C8—H8	120.2	H19A—C19—H19C	109.5
C7—C8—H8	120.2	H19B—C19—H19C	109.5
C8—C9—C10	120.9 (3)	C18—C20—H20A	109.5
C8—C9—H9	119.6	C18—C20—H20B	109.5
C10—C9—H9	119.6	H20A—C20—H20B	109.5
C5—C10—C9	120.4 (2)	C18—C20—H20C	109.5
C5—C10—H10	119.8	H20A—C20—H20C	109.5
C9—C10—H10	119.8	H20B—C20—H20C	109.5
N1—C11—C12	113.64 (14)	C18—C21—H21A	109.5
N1—C11—H11A	108.8	C18—C21—H21B	109.5
C12—C11—H11A	108.8	H21A—C21—H21B	109.5
N1—C11—H11B	108.8	C18—C21—H21C	109.5
C12—C11—H11B	108.8	H21A—C21—H21C	109.5
H11A—C11—H11B	107.7	H21B—C21—H21C	109.5
C17—C12—C13	117.77 (16)		
			
C2—N1—N2—C4	−0.45 (18)	C7—C8—C9—C10	−0.6 (4)
C11—N1—N2—C4	179.05 (14)	C6—C5—C10—C9	2.2 (3)
N2—N1—C2—C3	−0.09 (19)	C4—C5—C10—C9	−177.19 (19)
C11—N1—C2—C3	−179.51 (16)	C8—C9—C10—C5	−1.1 (4)
N2—N1—C2—C1	175.41 (15)	N2—N1—C11—C12	106.44 (18)
C11—N1—C2—C1	−4.0 (3)	C2—N1—C11—C12	−74.2 (2)
O2—C1—C2—N1	−4.8 (3)	N1—C11—C12—C17	120.67 (19)
O1—C1—C2—N1	177.14 (16)	N1—C11—C12—C13	−62.0 (2)
O2—C1—C2—C3	169.67 (19)	C17—C12—C13—C14	−1.0 (3)
O1—C1—C2—C3	−8.4 (3)	C11—C12—C13—C14	−178.41 (19)
N1—C2—C3—C4	0.56 (19)	C12—C13—C14—C15	0.8 (3)
C1—C2—C3—C4	−174.60 (17)	C13—C14—C15—C16	0.3 (3)
N1—N2—C4—C3	0.81 (19)	C13—C14—C15—C18	−178.8 (2)
N1—N2—C4—C5	−179.17 (14)	C14—C15—C16—C17	−1.0 (3)
C2—C3—C4—N2	−0.87 (19)	C18—C15—C16—C17	178.0 (2)
C2—C3—C4—C5	179.11 (16)	C13—C12—C17—C16	0.2 (3)
N2—C4—C5—C6	−160.76 (17)	C11—C12—C17—C16	177.62 (18)
C3—C4—C5—C6	19.3 (3)	C15—C16—C17—C12	0.8 (3)
N2—C4—C5—C10	18.6 (3)	C14—C15—C18—C19	95.8 (4)
C3—C4—C5—C10	−161.36 (19)	C16—C15—C18—C19	−83.2 (4)
C10—C5—C6—C7	−1.7 (3)	C14—C15—C18—C21	−133.1 (3)
C4—C5—C6—C7	177.74 (18)	C16—C15—C18—C21	47.9 (4)
C5—C6—C7—C8	0.0 (4)	C14—C15—C18—C20	−21.3 (4)
C6—C7—C8—C9	1.1 (4)	C16—C15—C18—C20	159.6 (3)

### Hydrogen-bond geometry (Å, °)

**Table:**

*D*—H···*A*	*D*—H	H···*A*	*D*···*A*	*D*—H···*A*
O1—H1···O2^i^	0.82	1.82	2.641 (2)	178

Symmetry codes: (i) −*x*+2, −*y*, −*z*+1.
